# Application of metagenomic next-generation sequencing in cutaneous tuberculosis

**DOI:** 10.3389/fcimb.2022.942073

**Published:** 2022-09-23

**Authors:** Min Kong, Wei Li, Qingsheng Kong, Haixin Dong, Aizhong Han, Liqing Jiang

**Affiliations:** ^1^ Affiliated Hospital of Jining Medical University, Jining Medical University, Jining, China; ^2^ Medical Laboratory of Jining Medical University, Jining Medical University, Jining, China

**Keywords:** tuberculosis, cutaneous tuberculosis, *mycobacterium tuberculosis* complex, metagenomic next-generation sequencing, pathogen

## Abstract

Tuberculous infection in a skin wound is a rare but well-known condition. This study describes a child infected with tuberculosis after being wounded. Because of swelling and pain in his wrist tissue, he was admitted to the Affiliated Hospital of Jining Medical University of Shandong Province on 16 October 2021. His medical history only included a wound. He was discharged after debridement. The laboratory data were normal. Two months after surgery, his wound was still swollen and painful. Secretions from the wound were sent for metagenomic next-generation sequencing (mNGS), which revealed three reads related to the *Mycobacterium tuberculosis* complex group (MTBC). A diagnosis of cutaneous tuberculosis (TB) was made. The wound disappeared after anti-TB drugs were administered. This case demonstrates that, while TB presenting as a severe cutaneous wound is rare, it should be considered in the clinical diagnosis. Clinicians should also pay attention to extrapulmonary infection with MTBC in patients, particularly in some long-suffering patients, and identify the specific pathogen as soon as possible. mNGS could help to identify pathogens and facilitate early treatment, thereby improving the prognosis.

## Introduction

Multiple epidemic diseases are affecting human health in China, including tuberculosis (TB). TB must be monitored and treated because of its extremely high death rate (Etter et al., 2006; Cardona, 2018). Among all types of TB, cutaneous TB is a rare form of extrapulmonary TB responsible for 1%–2% of all cases (Bravo and Gotuzzo, 2007; Ketata et al., 2015). *Mycobacterium tuberculosis* complex group (MTBC) usually enters the body through hangnail wounds, small wounds, impetigo, or furuncles (Hill and Sanders, 2017; Bespiatykh et al., 2021; Kanabalan et al., 2021). Its pathogenesis is similar to that of some other epidemic diseases (Issa et al., 2012). Over 2–4 weeks, *Mycobacterium* enters the body and slowly develops into a tuberculous chancre. The infection could progress to impetiginous or ichthyotic forms. Lymphatic extension occurs, and lymphadenopathy occurs 1–2 months after skin infection (Hill and Sanders, 2017; Tang et al., 2021).

Cutaneous TB is caused by members of MTBC (Sethuraman and Ramesh, 2013). Phylogenetically, MTBC is contained within the phylum Actinobacteria (Mostowy and Behr, 2005). Whole-genome sequencing has identified more than 20,000 strains of *Mycobacterium*, including 13,974 strains of *M*. *tuberculosis* complex, 13,896 strains of *M*. *tuberculosis* (Banuls et al., 2015), 69 strains of *M*. *canettii* (Supply and Brosch, 2017), 2 strains of *M*. *mungi* (Alexander et al., 2016), and 5 strains of *M*. *orygis* (Marcos et al., 2017). To date, the diagnostic method of TB has been traditional culture methods and multiple molecular approaches (Chen et al., 2020). Owing to the inherently slow growth rate of MTBC, conventional microbial culture results have an extremely low positivity rate. This increases the difficulty of clinical diagnosis and treatment. Therefore, rapid clarification of the etiology is necessary (Ma et al., 2022). Clinically, treatment is administered according to the doctor’s experience in most cases, leading to a higher possibility of the development of drug-resistant bacteria and the possible occurrence of secondary infections (Rimola et al., 2000; Grek and Arasi, 2016; Nanchal and Ahmad, 2016).

In recent decades, metagenomic next-generation sequencing (mNGS) has emerged as a rapidly developing new technology for etiological diagnosis (Jacob et al., 2019; Guo et al., 2021). In some infectious diseases (e.g., blood infection, central nervous system infection, lung infection, or focal infection), mNGS plays an important role in etiological diagnosis, especially in the identification of MTBC and rare pathogens, such as mycoplasma, *Chlamydia*, parasite, and viruses (Bezerra et al., 2020; Chen et al., 2020; Haston et al., 2020; Yu et al., 2020; Chen et al., 2021; Jing et al., 2021).

mNGS has the advantages of conventional culture and many other excellent properties (e.g., short turnaround times, high sensitivity and specificity) (Haslam, 2021). Furthermore, unrecorded pathogens can be initially detected by mNGS, along with the identification of non-culturable pathogens (Ren et al., 2021; Tang et al., 2021; Bohl et al., 2022). In the future, mNGS technology could become the primary method of identifying, predicting, and preventing infectious diseases (Han et al., 2019; Garnica et al., 2021; Yu et al., 2021).

This study explored the utility of mNGS for identifying pathogens in infected patients. Additionally, we explored the application of second-generation sequencing technology in wound infection.

## Case description

The case involved a 15-year-old Asian boy. Because of wrist swelling and painful discomfort after a bruise of the right wrist (on 30 September 2021), he was admitted to the Affiliated Hospital of Jining Medical University of Shandong Province, in China, on 16 October 2021.

The patient underwent fascial excision debridement to treat swelling and pain in the wrist tissue on 18 October 2021. Because the wound infection was alleviated and no exudation was noted, the patient was discharged on 31 October 2021. After discharge, the patient was reminded to regularly change the dressing and exercise.

However, the wound healed poorly, and there was still exudate 2 months later. The patient was again admitted on 9 December 2021 and underwent a second fascial excision debridement 2 days later. Samples of wound tissue were sent for microbial culture and mNGS (11 December 2021). The culture and mNGS failed to identify a pathogen. Infection-related indices, such as white blood cell count, C-reactive protein level, and neutrophilic granulocyte percentage, were normal although this was not true for procalcitonin. Thus, clinicians administered cefoperazone sodium as anti-infection therapy. One week later, most of the wounds on the right wrist had healed, except for a 3-mm wound in the middle of the wrist. Some exudate from the wound was present on the back of the right wrist (21 December 2021). Secretions from the wound were sent for mNGS, which revealed three reads for MTBC. Therefore, a diagnosis of cutaneous TB was considered. Accordingly, cefoperazone sodium administration was discontinued. Isoniazid (0.3 g/day), rifampicin (0.45 g/day), and pyrazinamide (1.5 g/day) were provided as anti-TB therapy for 3 months, and ethambutol (0.75 g) was provided as anti-TB therapy for one time. Re-examination of the wound on 29 January 2022 revealed that it had healed well. The clinicians recommended that the patient complete the course of anti-TB therapy.

The patient was in good condition during 3 months of follow-up. No significant abnormalities were found in the white blood cell count and C-reactive protein, procalcitonin, and uric acid levels in the serum.

## Materials and methods

### Specimen collection and processing

A 2.0- to 4.0-ml wound fluid sample (rinse solution) was collected from a debridement according to sample pretreatment instructions and inactivated in a water bath at 65°C for 30 min. A 1.5-ml microcentrifuge tube containing 480 μl of the sample and 72 U of lysozyme (Labnet VX-200, Xibao Biotechnology Co., Ltd., Shanghai, China) was placed in a metal bath at 30°C for 10 min. A screw tube containing the aforementioned sample and 250 μl of 0.5-mm glass beads was placed in a Labnet VX-200 oscillator set to the maximum oscillation for 20 min. Then, the sample was centrifuged at 8,000 rpm for 30 s, 300 μl of the supernatant was separated into a new 1.5-ml microcentrifuge tube, and DNA was extracted using a DNA Purification Kit (Huada Biotechnology Co., Ltd., Wuhan, China) according to the manufacturer’s instructions. A dsDNA HS Assay Kit 4.0 fluorometer was used to measure the DNA concentration, and DNA fragmentation was performed using a DNA enzyme digestion reaction kit (Huada Biotechnology Co., Ltd.) (Long et al., 2016).

### Preparation of DNA libraries and sequencing

DNA libraries were prepared using a PMseq™ High-throughput DNA Detection Kit (Huada Biotechnology Co., Ltd.). Processes, such as DNA fragmentation, repair, adapter ligation, and unbiased polymerase chain reaction (PCR) amplification, were performed strictly in accordance with the instructions. A Qubit dsDNA HS Assay Kit 4.0 fluorometer was used to measure the DNA concentration in each sample. The quality-qualified libraries were pooled in a 0.2-ml PCR tube. A MiSeq™ Dx Reagent Kit (Huada Biotechnology Co., Ltd.) was used to prepare the DNA nanoball. Finally, the samples were sequenced using the Huada mNGS platform (Jeon et al., 2014).

### Sequencing analysis and determination of pathogens

Some data, such as those for adapter contamination and low-quality and low-complexity reads, were quality-filtered. Next, Burrows-Wheeler Alignment (Version 0.7.10) was used to map the filtered sequences to a human reference database (Li and Durbin, 2009). The rest of the data were assembled using PMSEQ bioinformatics analysis software and classified *via* simultaneous alignment to the pathogen metagenomics database (PMDB) containing 17,500 pathogens. To date, the PMDB contains 10,989 bacterial genomes or scaffolds (196 *Mycobacterium* and 159 *Mycoplasma*, *Chlamydia*, and *Rickettsia*), 1,179 fungi related to human diseases, 5,050 whole-genome sequences of viral taxa, and 282 parasites associated with human infection (Chen et al., 2021; Yu et al., 2021; Ma et al., 2022).

The read counts and genomic coverage were classified and recorded. After receiving the results of taxonomic assignments, we aligned reads mapped to MTBC by MegaBLAST to the PMDB pathogen metagenomics database with default parameters for further confirmation. For example, the mNGS result was positive when the number of bacterial or viral reads was 10-fold greater than that of any other microbes and when the number of fungi (species level) was 5-fold greater than that of other fungi. A positive result for mNGS was given as long as the number of MTBC reads at the genus level was only 1 (Ma et al., 2022). The stringent mapped reads number (SMRN) and genomic coverage (GC) were also used as references. SMRN refers to the number of reads that are strictly mapped to the genus of the pathogen, and it can reflect the sequencing depth of the detected pathogens to some extent. Multiple factors (e.g., content of pathogen in the sample, the size of the pathogen genome, the amount of DNA extracted from the sample) affect SMRN. A higher SMRN indicates a greater likelihood that the detected microbe is pathogenic. GC refers to the percentage of the DNA sequence length of the detected pathogen that matches the known genomic sequence of the pathogen. Generally speaking, a higher GC indicates greater credibility regarding the detected pathogen. However, GC is also influenced by the type and content of the pathogen (Tang et al., 2021).

## Results and discussion

TB is a pandemic disease that typically presents with clinical symptoms including fever, cough of any duration, night sweats, and weight loss (Burke et al., 2022). Currently, pathogen detection methods and techniques (e.g., traditional culture methods and multiple molecular approaches) are the predominant options for diagnosing TB (Austin, 2017).

Among TB types, cutaneous TB is frequently found, especially in developing and tropical countries (Padmavathy et al., 2007). Wound infection is one of the most serious complications of trauma. Most patients have open wounds caused by accidents. Long-term exposure to the air makes them vulnerable to contamination by pathogenic bacteria in the environment, resulting in wound infection. Cutaneous TB is a rare infectious disease. It can present with some common clinical manifestations (e.g., lupus vulgaris, scrofuloderma, TB verrucosa cutis, orificial TB, tuberculous gumma, tuberculous chancre, and acute cutaneous miliary TB) (Santos et al., 2014). However, some studies reported that the clinical manifestations are not specific in most cases of TB. To date, culture and acid-fast bacilli tests are the gold standards for TB diagnosis. TB bacilli should be easily detected in specimens; however, few or no TB bacilli can be detected because of the difficulty of culture (Vashisht et al., 2007).

**Figure 1:**
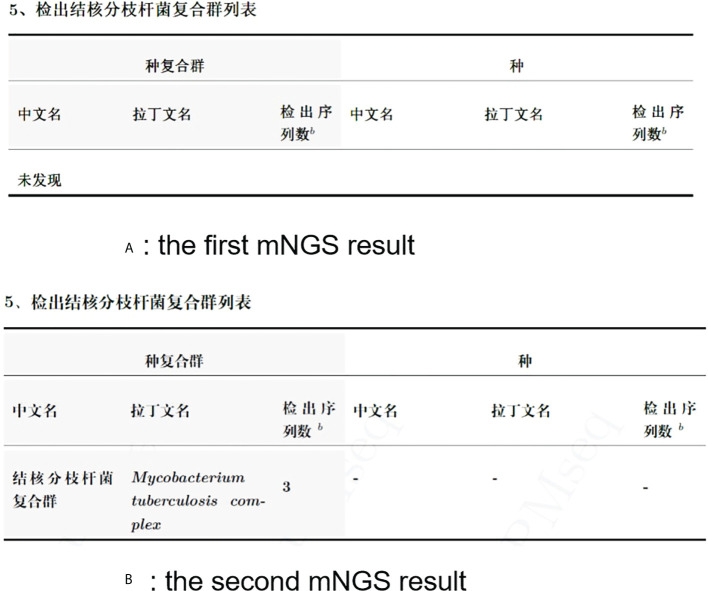
**(A)** The first mNGS result. **(B)** The second mNGS result.

In this study, the patient underwent debridement surgery. The laboratory findings from the patient (white blood cells, 4.92; C-reactive protein, 0.25; procalcitonin, 0.055; erythrocyte sedimentation rate, 1.00) were almost normal. No pathogen was detected in the culture and smear. However, the postoperative healing was poor. In addition, targeted medication could not be used without an etiological basis. Two months later, the patient underwent another debridement surgery. The wound secretion of the patient was again negative in culture, and his laboratory findings were normal. However, MTBC was detected by mNGS (SMRN, 3; GC, 54/4,318, 603). The two results obtained by mNGS are presented in **Figure 1**. The patient improved after treatment with anti-TB drugs (rifampicin, pyrazinamide, isoniazid, and ethambutol) for 3 months. On 3 May 2022, anti-TB therapy has since been discontinued.

The difficulty of detecting the pathogenic cause of cutaneous TB is difficult for several reasons. First, tuberculous bacilli have a long growth cycle, and they are difficult to culture. Second, the patient was already on medication before pathogen identification, thereby increasing the chances of a negative culture. Finally, mNGS has significantly better sensitivity and specificity for detecting pathogenic bacteria than culture.

In June 2020, Chinese experts published an expert consensus for the application of China’s mNGS technology in pathogen detection in moderate and severe clinical infections (Consensus Group of Experts on Application of Metagenomic Next Generation Sequencing in the Pathogen Diagnosis in Clinical, Moderate, Severe, Infections, Professional Committee Of, Sepsis, Shock Chinese Research Hospital, Association, Professional Committee of Microbial Toxins Chinese Society For, Microbiology, and Professional Committee of Critical Care Medicine Shenzhen Medical, Association, 2020). This statement described the expert consensus regarding etiological diagnosis based on mNGS in the scope of detection, rules, and applications in moderate and severe clinical infections and highlighted the future improvement of its clinical application.

Several studies have reported the ability of mNGS to detect pathogens (Xia et al., 2019; Liu et al., 2021; Zhao et al., 2021). For example, clinicians suspected bacterial infection even though conventional microbiological tests were negative. Meanwhile, a positive result was obtained *via* mNGS, illustrating its greater utility for detecting pathogens (e.g., *herpesvirus type 6*, *Pneumocystis jirovecii*, or *Stenotrophomonas maltophilia*) in some cases. In addition, mNGS can be used to exclude fever as a sign of infection and reduce the abuse of antibiotics in infected patients (Zinner, 2007; Tang et al., 2021).

However, mNGS has limitations (Filkins et al., 2020). First, there is no uniform standard experimental procedure, and it is difficult to distinguish between pathogens and colonizing bacteria (Filkins et al., 2020). Second, the use of antibiotics can affect the diagnostic results of mNGS. Third, RNA is generally not sequenced in patient samples because of its high cost; therefore, infections caused by RNA viruses (e.g., human immunodeficiency virus or influenza A virus) may be partially undetected (Chiu and Miller, 2019). Fourth, it is especially difficult to differentiate contaminants, colonizers, and pathogens using mGNS in the case of severe infections. Finally, the cost of mNGS can be prohibitive to patients. It is not conducive to the development of clinical work and cannot help in learning how mNGS can be implemented in real-world clinical settings.

In conclusion, the findings suggest that mNGS is superior to traditional laboratory testing methods, and this method can be performed more rapidly. We believe that mNGS will soon become a candidate method for clinical diagnosis in patients infected with TB. mNGS will likely be used for the rapid detection of pathogens in the future, particularly in the context of opportunistic pathogens and mixed infections or in patients with negative conventional microbiological test results. Therefore, to better serve the clinic, we must clarify how to identify pollutants, colonizers, and pathogens in the future.

## Data availability statement

The raw data supporting the conclusions of this article will be made available by the authors, without undue reservation.

## Ethics statement

Written informed consent was obtained from the individual(s) for the publication of any potentially identifiable images or data included in this article.

## Author contributions

MK and AH performed the experiments and analyzed the genomics data. WL analyzed and interpreted patient data. MK, QK, HD, and LJ wrote the manuscript. All authors read and approved the final manuscript.

## Funding

This work was supported by the Medical Laboratory of Jining Medical University, Jining Medical University.

## Conflict of interest

The authors declare that the research was conducted in the absence of any commercial or financial relationships that could be construed as a potential conflict of interest.

## Publisher’s note

All claims expressed in this article are solely those of the authors and do not necessarily represent those of their affiliated organizations, or those of the publisher, the editors and the reviewers. Any product that may be evaluated in this article, or claim that may be made by its manufacturer, is not guaranteed or endorsed by the publisher.

## Abbreviations

MTBC, *Mycobacterium tuberculosis* complex; TB, tuberculosis; mNGS, metagenomic next-generation sequencing.
